# Demographic and clinical profile of residents living with dementia and depressive symptoms in Australian private residential aged care: Data from the Music Interventions for Dementia and Depression in ELderly care (MIDDEL) cluster‐randomised controlled trial

**DOI:** 10.1111/ajag.13104

**Published:** 2022-07-08

**Authors:** Young‐Eun C. Lee, Tanara Vieira Sousa, Phoebe A. Stretton‐Smith, Christian Gold, Monika Geretsegger, Felicity A. Baker

**Affiliations:** ^1^ Creative Arts and Music Therapy Research Unit Faculty of Fine Arts and Music The University of Melbourne Melbourne Victoria Australia; ^2^ Psychology and Specialist Services, Monash Health Melbourne Victoria Australia; ^3^ NORCE Norwegian Research Centre AS Bergen Norway; ^4^ Centre for Music and Health Norwegian Academy of Music Oslo Norway

**Keywords:** Australia, dementia, depression, quality of life

## Abstract

**Objectives:**

1) To describe the demographic and clinical characteristics of residents with dementia and depressive symptoms in the Australian private residential aged care (RAC) context; and 2) to investigate the association between neuropsychiatric symptoms, depression and quality of life and their interactions with dementia severity.

**Methods:**

This study examined the baseline demographic and clinical data from the Australian arm of the Music Interventions for Dementia and Depression in ELderly care (MIDDEL) study, a multinational, cluster‐randomised controlled trial. Demographic characteristics, neuropsychiatric symptoms, depression, quality of life and dementia severity were collected in 330 residents of 12 private RAC facilities across Melbourne, Australia. Descriptive statistics, the Kruskal–Wallis test and the Pearson Χ^2^ test were used to describe and compare the demographic and clinical characteristics according to dementia severity. The association between clinical characteristics and dementia severity was examined using linear regression analyses.

**Results:**

Residents' mean age was 86.5 years, 69% were female, and 44.2% had severe dementia. There were no significant differences between the dementia severity groups on age, sex and education. Residents with severe dementia were more likely to have a diagnosis of Alzheimer's disease (40.3%) and be born overseas (46.8%). Higher levels of neuropsychiatric symptoms, distress and depressive symptoms, and lower quality of life were associated with more severe dementia.

**Conclusions:**

The findings from our study highlight the diverse and complex care needs of people living with dementia in the Australian private RAC setting, which can be used to inform targeted, person‐centred dementia care planning, staff training and allocation of resources.

**Policy Impact**

This study found that higher levels of neuropsychiatric symptoms and depression, and lower quality of life were associated with higher severity of dementia in Australian private residential aged care residents with dementia and depressive symptoms. This suggests that care models and resources should focus on meeting social and emotional needs in addition to medical and physical needs.

**Practice Impact**

A high proportion of residents with severe dementia living in residential aged care facilities were from culturally and linguistically diverse backgrounds. Professional carers should be trained to deliver culturally safe care that focuses on social and emotional needs in addition to medical and physical needs.

## 1 INTRODUCTION

In Australia, there are currently over 244,000 people living in permanent residential aged care (RAC).^1^ Dementia is common among RAC residents, with recent data suggesting that approximately half (54%) of people living in RAC have a diagnosis of dementia.^1^ RAC is a common pathway for people with dementia in comparison with those without dementia, possibly reflecting their high care needs.

A key component of providing high‐quality care for people with dementia is a person‐centred approach, which requires a detailed understanding of individuals' key demographic characteristics and care needs within the RAC setting. Further, determining the pattern and factors associated with dementia severity enables researchers to develop interventions tailored to specific subgroups of people with dementia to target key areas of needs and the management of resources; it is the first step to develop and improve dementia care service provision in the RAC setting.^2^

Neuropsychiatric symptoms are common in residents with dementia residing in RAC settings, with a prevalence ranging between 82 and 92% during the course of disease.^3^ Neuropsychiatric symptoms are a heterogeneous group of non‐cognitive symptoms and behaviours, including depression, anxiety, apathy, delusions, hallucinations and sleep impairment. When these symptoms occur in the context of dementia, they are independently associated with poor outcomes, including increased morbidity, mortality, hospital stays, and accelerated functional and cognitive decline in addition to challenges associated with caregiving and a significant rise in the cost of care.^4^

In recent years, there has been increasing interest in examining the relationship between dementia severity and a range of clinical outcomes, such as neuropsychiatric symptoms and quality of life. Cross‐sectional and longitudinal studies involving community‐dwelling people with Alzheimer's disease (AD) have demonstrated an increase in neuropsychiatric symptoms with the progression of AD, which is associated with a decline in caregiver‐rated quality of life.^5^, ^6^ This is consistent with other research finding neuropsychiatric symptoms, such as agitation, disinhibition and irritability, to be more frequent in the later stages of dementia in community‐dwelling people with AD, vascular dementia (VaD) and frontotemporal dementia (FTD).^7^

In the RAC context, a number of studies have examined the relationship between neuropsychiatric symptoms with cognitive impairment and functional status in dementia.^8^, ^9^ In a large cohort of Dutch RAC residents, Zuidema and colleagues found that the prevalence of neuropsychiatric symptoms was associated with moderately severe cognitive decline, while specific symptoms such as physical aggression, anxiety and apathy were more frequent with very severe cognitive decline.^8^ A more recent cross‐sectional cohort study in RAC facilities in Helsinki showed that the severity of neuropsychiatric symptoms and dementia severity were significant factors determining health‐related quality of life.^9^ In this study, higher scores in neuropsychiatric symptoms were associated with better quality of life in residents with severe dementia, while this association was not observed for mild‐to‐moderate dementia. The authors suggested that the differences may be partly due to study population, due to the inclusion of a higher proportion of residents with severe dementia (67%) in their study compared to previous studies. Taken together, these findings indicate that neuropsychiatric symptoms and quality of life may have a distinct impact at different stages of dementia. However, there is a scarcity of research examining the relationship between severity and specific subdomains of neuropsychiatric symptoms, quality of life and dementia severity in the Australian RAC context.

Using a prospective cohort of older people with dementia and mild‐to‐severe depressive symptoms living in RAC who were part of a large clinical trial, the aim of our study was to: 1) describe the demographic and clinical characteristics of people with dementia residing in private RAC facilities across the Melbourne metropolitan area, Australia; and 2) investigate the cross‐sectional association between neuropsychiatric symptoms, depression and quality of life, and their interactions with the severity of dementia.

## 2 METHODS

### 2.1 Design and setting

This cross‐sectional study used baseline data from Music Interventions for Dementia and Depression in ELderly care (MIDDEL), a multinational cluster‐randomised controlled trial being conducted in RAC in Australia, Germany, the Netherlands, Norway, Turkey and the United Kingdom. Australia was the first country to start implementation, and completed data collection in January 2021, prior to other countries commencing study implementation. Details of the study protocol and main findings are described elsewhere.^10^, ^11^ Participants were recruited from 12 private RAC facilities in Melbourne, Australia.

### 2.2 Sample

The study sample included residents who met the following inclusion criteria: ≥65 years of age; dementia as indicated by a Clinical Dementia Rating (CDR)^12^ score of 0.5 to 3; a Mini‐Mental State Examination (MMSE)^13^ score of ≤26; and mild‐to‐severe depression symptoms indicated by a Montgomery–Åsberg Depression Rating Scale (MADRS)^14^ score of >8. Residents were excluded if they were in short‐term care, with known diagnoses of schizophrenia or Parkinson's disease, severe hearing impairment, or unable to tolerate sitting in a chair for at least part of the sessions that were provided as part of the trial.

### 2.3 Ethical considerations

Ethics approval was obtained from the Medicine and Dentistry Human Ethics Sub‐Committee at the University of Melbourne, Australia (12 January 2018, Ethics ID: 1750400). All participants gave informed written consent or assent to participate in the study. For those residents not able to provide consent, their guardian, next of kin or significant other provided consent on their behalf. All study procedures were conducted in accordance with the ethical principles for involving people with cognitive impairment in research studies that are outlined in the Australian National Statement for Ethical Conduct in Human Research.

### 2.4 Measures

The main outcomes for answering the research questions were neuropsychiatric symptoms, depression and quality of life measures. Neuropsychiatric symptoms were assessed using the Neuropsychiatric Inventory Questionnaire (NPI‐Q)^15^ completed by professional care staff most familiar with participating residents. The NPI‐Q is the most highly regarded and widely used measure for determining neuropsychiatric symptoms in clinical trials and is translated into more than 40 languages. It has been cross‐validated against the NPI as the gold standard (*r* = 0.73) and has demonstrated good validity (sensitivity = 74.1%, specificity = 79.5%), reliability (Cronbach's alpha = 0.78) and excellent test–retest reliability (*r =* 0.99).^15^ The NPI‐Q comprises 12 symptom domains rated for symptom severity (1—mild; 2—moderate; and 3—severe; total scores ranging from 0 to 36), and the associated distress of symptoms for caregivers (from 0 (not distressing at all) to 5 (extreme or very severe); total scores ranging from 0 to 60). In addition, we explored the NPI‐Q subscales validated by Trzepacz and colleagues^16^: i) NPI‐Q‐4‐Agitation/aggression comprises the items ‘agitation/aggression’, ‘disinhibition’, ‘irritability/lability’ and ‘motor disturbance’ (score ranging from 0 to 12); ii) NPI‐Q‐3‐Mood includes ‘depression/dysphoria’, ‘anxiety’ and ‘irritability/lability’ (score ranging from 0 to 9); and iii) NPI‐Q‐4‐Frontal includes ‘elation/euphoria’, ‘apathy/indifference; ‘disinhibition’ and ‘irritability/lability’ (score ranging from 0 to 12).

Levels of depressive symptoms were measured using the MADRS.^14^ The MADRS is a clinician‐rated 10‐item scale; each item is rated from 0 (no abnormality) to 6 (severe), and total scores range from 0 to 60, with higher scores indicating higher severity of depressive symptoms. The MADRS has been used successfully in studies with people with dementia. It has shown high reliability and validity and is sensitive to change in comparison with alternative scales, such as the Cornell Scale for Depression in Dementia (CSDD).^17^ For MADRS, we also explored four subdomains or ‘factors’, as proposed by Williamson^18^: i) sadness factor comprises the items ‘apparent sadness’ and ‘reported sadness’; ii) neurovegetative factor includes ‘inner tension’, ‘reduced sleep’ and ‘reduced appetite’; iii) detachment factor includes ‘concentration difficulties’, ‘lassitude’ and ‘inability to feel’; and iv) negative thoughts factor includes ‘pessimistic thoughts’ and ‘suicidal thoughts’. Each factor ranges from 0 to 6 and is an average of the domain scores.

Quality of life was assessed using the EuroQol (EQ‐5D‐5L),^19^ which evaluates the health‐related quality of life. The EQ‐5D‐5L has two parts: a descriptive system and a visual analogue scale.^17^ The EQ‐5D descriptive system assesses health in five dimensions (mobility, self‐care, usual activities, pain or discomfort, and anxiety or depression) and on five levels (from 1 (no problem) to 5 (severe problems)). For this study, we used an Australian value set crosswalk to present a social perspective through the weighted index. The EQ‐VAS^19^ (visual analogue scale) assesses perceived health, rated from 0 to 100 (0 (the worst) to 100 (the best imaginable health)). For this study, the EQ‐5D‐5L was assessed by the residents' proxy (professional care staff). Previous studies indicate that the selection of assessment mode and the choice of appropriate proxies are important to ensure validity in studies of people with dementia.^20^

To examine the association between neuropsychiatric symptoms, depression, quality of life and their interactions with dementia severity, we categorised CDR ratings into three categories for this analysis: very mild/mild (CDR 0.5–1), moderate (CDR 2) and severe (CDR 3). Potentially confounding variables included demographic characteristics, such as sex, age, marital status, country of birth, language and education. MMSE was not included as a confounder variable because of its correlation with CDR (Spearman’s rho = −0.826).

### 2.5 Statistical analysis

Descriptive statistics were calculated and presented by mean and standard deviation, absolute number and percentage, as appropriate. To describe the characteristics of this population according to the severity of dementia, the Kruskal–Wallis test and the Pearson Χ^2^ test were presented for continuous and categorical variables, respectively. We used a non‐parametric test for non‐categorical variables, after checking for normality using a histogram and the Kolmogorov–Smirnov test.

To test the association between neuropsychiatric symptoms, depression and quality of life with the severity of dementia, linear regression analyses were performed for each main outcome (NPI‐Q severity, NPI‐Q distress, MADRS, EQ‐5D‐5L and VAS). We presented the results of two models: unadjusted and adjusted to the demographic characteristic variables.

Multicollinearity tests were undertaken between independent variables using the variance inflation factor (VIF). Analysis was conducted using STATA v16.1 (StataCorp, College Station, TX, USA).

## 3 RESULTS

Between June 2018 and January 2020, we screened 818 residents across 12 private Australian RAC facilities. Of these residents, 330 residents agreed to participate in the MIDDEL trial and completed baseline assessments, which represent the data pool for this study. The demographic and clinical variables of all participants, stratified by CDR groups, are shown in Table 1. There were 69 residents in CDR 0.5–1 (mild), 115 residents in CDR 2 (moderate) and 146 in CDR 3 (severe).

**TABLE 1:** Music Interventions for Dementia and Depression in ELderly care (MIDDEL)—Residents' characteristics by CDR at Baseline

	Total	CDR 0.5–1	CDR 2	CDR 3	*p* value
Assessed at baseline, *n* (%)	330	69 (20.9)	115 (34.9)	146 (44.2)	
Age, mean (SD)	86.5 (7.3)	86.6 (7.1)	85.9 (7.5)	86.9 (7.2)	0.675
Female, *n* (%)	229 (69.4)	47 (68.1)	77 (67.0)	105 (71.9)	0.666
Marital status, *n* (%)					
Married	101 (31.1)	17 (25.0)	27 (23.7)	57 (40.1)	0.025*
Single/separated/divorced	47 (14.6)	14 (20.6)	18 (15.9)	15 (10.6)	
Widow/widower	175 (54.2)	37 (54.4)	68 (60.2)	70 (49.2)	
Country of birth, *n* (%)					
Australia	193 (59.4)	49 (72.1)	69 (60.0)	75 (52.8)	0.033*
Other countries	131 (40.4)	19 (27.9)	46 (40.0)	66 (46.8)	
First language, *n* (%)					
English	239 (75.9)	59 (86.8)	82 (71.9)	98 (71.5)	0.006**
Other language (good knowledge of English)	51 (16.0)	9 (13.2)	23 (20.2)	19 (13.9)	
Other language (poor/no knowledge of English)	29 (9.1)	0	9 (7.9)	20 (14.6)	
Highest level of education completed, *n* (%)					
Primary education	70 (21.6)	13 (19.1)	22 (19.5)	35 (24.5)	0.409
Secondary education	172 (53.1)	35 (51.5)	66 (58.4)	71 (49.7)	
Tertiary/further education	40 (12.4)	11 (16.2)	15 (13.3)	14 (9.8)	
Not known	42 (13.0)	9 (13.2)	10 (8.9)	23 (16.1)	
Clinical diagnosis of dementia, *n* (%)					
Alzheimer's disease	107 (34.1)	10 (15.9)	39 (36.5)	58 (40.3)	0.008**
Other dementia types	60 (19.1)	12 (19.1)	21 (19.6)	27 (19.9)	
Unspecified dementia	147 (46.8)	41 (65.1)	47 (43.9)	59 (41.0)	
MMSE, mean (SD)	8.1 (7.8)	18.7 (4.8)	9.7 (4.7)	1.8 (3.6)	<0.001***
MADRS total score, mean (SD)	18.2 (7.5)	13.5 (4.7)	16.1 (6.1)	22.2 (7.8)	<0.001***
MADRS factors, mean (SD)					
Sadness	2.4 (1.4)	2.4 (0.9)	2.4 (1.2)	2.5 (1.8)	0.544
Neurovegetative	1.5 (1.0)	1.1 (0.7)	1.4 (0.9)	1.7 (1.1)	0.002**
Detachment	2.7 (1.6)	1.6 (1.0)	2.1 (1.0)	3.8 (1.5)	<0.001***
Negative thoughts	0.3 (0.7)	0.3 (0.6)	0.4 (0.7)	0.3 (0.8)	0.613
NPI‐Q severity, mean (SD)	10.7 (6.7)	7.7 (4.6)	9.7 (5.8)	13 (7.5)	<0.001***
NPI‐Q distress, mean (SD)	12.0 (10.9)	9.1 (7.6)	11.2 (9.7)	13.9 (12.7)	0.0902
NPI‐Q subscales, mean (SD)					
NPI‐Q‐4‐Agitation/aggression	3.8 (3.4)	2.5 (2.6)	3.2 (3.0)	4.9 (3.7)	<0.001***
NPI‐Q‐3‐Mood	4.2 (2.3)	3.7 (1.9)	4.2 (2.3)	4.5 (2.6)	0.122
NPI‐Q‐4‐Frontal	3.4 (2.8)	2.5 (2.1)	3.0 (2.6)	4.1 (3.1)	<0.001***
EQ‐5D‐5L (AUS‐weighted) mean (SD)	0.362 (0.30)	0.523 (0.29)	0.460 (0.27)	0.210 (0.25)	<0.001***
EQ‐5D‐VAS	55.0 (18.3)	63.5 (16.9)	56.9 (14.8)	49.3 (19.7)	<0.001***

*Note*: For continuous variables, we used the Kruskal–Wallis test to determine the difference between groups. For categorical variables, we used the Pearson Χ^2^ test. *p < 0.05; **p < 0.01; and ***p < 0.001. CDR, Clinical Dementia Rating—0.5‐1 (very mild to mild), 2 (moderate) and 3 (severe); MMSE, Mini‐Mental State Examination; MADRS, Montgomery–Åsberg Depression Rating Scale; NPI‐Q, Neuropsychiatric Inventory Questionnaire; EQ‐5D‐5L^AUS^, EuroQol health‐related quality of life 5 dimensions, 5 levels, adjusted by societal weights using the Australian crosswalk value set estimated by the authors; and EQ‐5D‐VAS, EuroQol visual analogue scale.

### 3.1 Demographic characteristics

The mean age of the residents was 86.5 years, 69% were female, and 44.2% had severe dementia (CDR = 3). Approximately half (53.1%) had completed secondary education, with a mean MMSE score of 8.1. The three CDR groups were similar in demographic characteristics, including age, sex and education. In contrast, the groups differed significantly in the country of birth and first language. More residents in the severe dementia group were born overseas (46.8%), compared with 27.9% in the mild dementia group. Moreover, the severe dementia group consisted of significantly more married participants (40.1%) in comparison with 25.0% and 23.7% in the mild dementia group and the moderate dementia group, respectively (Table 1).

### 3.2 Clinical characteristics

Significant differences were noted in clinical characteristics. Residents with severe dementia were significantly more likely to have a diagnosis of AD (40.3%) than other dementia diagnoses, whereas residents in the mild dementia group were significantly more likely to have a diagnosis of unspecified dementia. There were significant group differences in MADRS and NPI‐Q severity with residents in the severe dementia group, demonstrating a higher level of symptoms on these outcomes. Residents in the severe dementia group demonstrated a poorer quality of life as assessed by the EQ‐5D‐5L and VAS.

### 3.3 Neuropsychiatric symptoms and dementia severity

As shown in Table 2, NPI‐Q severity and NPI‐Q distress were significantly influenced by dementia severity in the linear regression analysis, with the highest levels appearing in residents with severe dementia (CDR 3): a severity of 5.56 (*p* < 0.001) points higher compared with residents with mild dementia. Similarly, NPI‐Q‐4‐Agitation/aggression and NPI‐Q‐3‐Frontal subscales showed the highest symptoms in residents with severe dementia: 2.56 (*p* < 0.001) and 1.81 (*p* < 0.001) points compared to residents in the beginning of their dementia journey. These findings were robust after adjusting for demographic variables (age, education, sex and marital status).

**TABLE 2:** Association between neuropsychiatric symptoms (NPI‐Q) and dementia severity

	Unadjusted			Adjusted to demographic characteristics		
	β	*p* value	95% CI	β	*p* value	95% CI
NPI‐Q severity						
CDR moderate	1.99	0.046*	0.04 to 3.93	1.93	0.056	−0.05 to 3.91
CDR severe	5.33	<0.001***	3.45 to 7.2	5.56	<0.001***	3.63 to 7.50
NPI‐Q distress						
CDR moderate	2.05	0.219	−1.22 to 5.33	1.72	0.317	−1.66 to 5.10
CDR severe	4.78	0.003**	1.61 to 7.95	4.32	0.010**	1.02 to 7.63
NPI‐Q‐4‐Agitation/aggression						
CDR moderate	0.72	0.151	−0.26 to 1.70	0.63	0.211	−0.36 to 1.61
CDR severe	2.39	<0.001***	1.44 to 3.34	2.56	<0.001***	1.61 to 3.52
NPI‐Q‐3‐Mood						
CDR moderate	0.51	0.158	−0.20 to 1.21	0.51	0.167	−0.21 to 1.23
CDR severe	0.73	0.036*	0.05 to 1.41	0.82	0.025*	0.11 to 1.52
NPI‐Q‐4‐Frontal						
CDR moderate	0.44	0.304	−0.40 to 1.27	0.44	0.302	−0.40 to 1.28
CDR severe	1.59	<0.001***	0.78 to 2.39	1.81	<0.001***	0.99 to 2.62

*Note*: *p < 0.05; **p < 0.01; and ***p < 0.001. NPI‐Q, Neuropsychiatric Inventory Questionnaire; CDR, Clinical Dementia Rating; CDR moderate = 2, CDR severe = 3; and CDR mild (0.5–1) as base case. Results were adjusted to age, sex, marital status, country of birth and education.

### 3.4 Depression and dementia severity

The results in Table 3 show the relationship between the MADRS total score and dementia severity. A higher MADRS total score was associated with severe dementia. In particular, neurovegetative and detachment factors on MADRS showed highest levels in residents with severe dementia. These findings were robust after adjusting for demographic variables.

**TABLE 3:** Association between depression (MADRS) and dementia severity

	Unadjusted			Adjusted to demographic characteristics		
	β	*p* value	95% CI	β	*p* value	95% CI
MADRS total score						
CDR moderate	2.60	0.012*	0.58 to 4.61	2.82	0.009**	0.72 to 4.92
CDR severe	8.72	<0.001***	6.77 to 10.67	8.83	<0.001***	6.77 to 10.88
Sadness						
CDR moderate	−0.04	0.862	−0.46 to 0.39	−0.06	0.807	−0.49 to 0.39
CDR severe	0.15	0.465	−0.36 to 0.56	0.11	0.622	−0.33 to 0.54
Neurovegetative						
CDR moderate	0.26	0.098	−0.05 to 0.56	0.30	0.0964	−0.02 to 0.62
CDR severe	0.56	<0.01**	0.27 to 0.85	0.57	<0.01**	0.26 to 0.88
Detachment						
CDR moderate	0.58	0.003**	0.20 to 0.96	0.63	0.001**	−0.25 to 1.02
CDR severe	2.21	<0.001***	1.85 to 2.58	2.25	<0.001***	1.88 to 2.63
Negative thoughts						
CDR moderate	0.09	0.429	−0.13 to 0.31	0.06	0.581	−0.16 to 0.30
CDR severe	0.05	0.657	−0.16 to 0.26	0.07	0.553	−0.16 to 0.29

*Note*: *p < 0.05; **p < 0.01; and ***p < 0.001. MADRS, Montgomery–Åsberg Depression Rating Scale; CDR, Clinical Dementia Rating; CDR moderate = 2, CDR severe = 3; and CDR mild (0.5–1) as base case.

### 3.5 Quality of life and dementia severity

As shown in Table 4, EQ‐5D‐5L and VAS scores were significantly associated with dementia severity, with a decrease in both appearing in residents with severe dementia (CDR 3). For residents with moderate dementia (CDR 2), only VAS was significantly lower compared to mild dementia (−6.63, *p* = 0.015). These findings were robust after adjusting for demographic variables.

**TABLE 4:** Association between the quality of life (EQ‐5D‐5L and VAS) and dementia severity

	Unadjusted			Adjusted to demographic characteristics		
	β	*p* value	95% CI	β	*p* value	95% CI
EQ‐5D‐5L^AUS^						
CDR moderate	−0.06	0.124	−0.14 to 0.02	−0.05	0.225	−0.14 to 0.03
CDR severe	−0.31	<0.001***	−0.39 to −0.24	−0.30	<0.001***	−0.38 to −0.22
EQ‐5D‐VAS						
CDR moderate	−6.63	0.015*	−11.97 to −1.27	−6.07	0.030*	−11.56 to −0.59
CDR severe	−14.18	<0.001***	−19.35 to −9.01	−13.06	<0.001***	−18.4 to −7.68

*Note*: *p < 0.05; **p < 0.01; and ***p < 0.001. EQ‐5D‐5L^AUS^, EuroQol health‐related quality of life 5 dimensions, 5 levels, adjusted by societal weights using the Australian crosswalk value set estimated by the authors; EQ‐5D‐VAS, EuroQol visual analogue scale; CDR, Clinical Dementia Rating; CDR moderate = 2, CDR severe = 3; and CDR mild (0.5–1) as base case.

## 4 DISCUSSION

This study examined the clinical and demographic characteristics and the association between severity of dementia with neuropsychiatric symptoms and quality of life in residents with dementia and mild‐to‐severe depressive symptoms in the Australian private RAC context. We found that the demographic and clinical profile of the residents in our study was similar to that of the older Australians residing in RAC reported in previous literature.^21^

Our study found that among residents with dementia and depressive symptoms, the severity and caregiver distress associated with neuropsychiatric symptoms were associated with the severity of dementia. In particular, agitation, aggression, irritability, disinhibition, euphoria and apathy scores were higher in those with increased dementia severity. Similar findings have been reported in previous studies examining people with different types of dementia (AD, VaD and FTD) in community^6^, ^7^ and RAC contexts.^8^, ^9^ Findings from our study also showed that dementia severity was negatively associated with the quality of life. This is consistent with two systematic reviews that noted a negative association between caregiver‐rated quality of life and dementia severity.^22^, ^23^ The association between higher dementia severity and poor quality of life has also been reported by a recent cross‐sectional cohort study in RAC in the Netherlands,^24^ and in longitudinal studies.^6^

In our study, more severe dementia (CDR ≥2) was associated with a higher level of depressive symptoms. In addition, we found that a higher level of neurovegetative and detachment symptoms (reduced appetite, reduced sleep, concentration difficulties, lassitude and inability to feel) was related to the severity of dementia. This is in contrast to a previous study of residents in RAC facilities that reported a decreasing prevalence of depression with the increasing severity of dementia.^8^ This discrepancy may be related to the differences in measures used and the broader inclusion of residents with more severe dementia in our study. Further, these differences may be due to our inclusion of residents with dementia and mild‐to‐moderate depressive symptoms (mean MADRS = 18.2).

Our findings are relevant in the context of the recent Australian Royal Commission into Aged Care,^25^ which suggested a lack of access to appropriate services due to limited understanding of the complex care needs of residents with dementia. In particular, the Royal Commission Final Report highlighted that the current care model in RAC focuses on meeting the medical and physical needs of residents rather than proactively supporting residents' social and emotional needs. A detailed understanding of the care needs of residents with dementia can aid the ability for care staff to deliver appropriate dementia care. In this study, we characterised the diverse needs and progression of neuropsychiatric symptoms in residents with dementia and depressive symptoms living in Australian private RAC facilities. Our findings can be used in the training of staff to support an understanding of the changing psychosocial needs of residents with dementia, to inform care planning and to improve the model of care for residents at different stages of dementia. Moreover, our findings highlight the additional needs of residents with dementia from culturally and linguistically diverse backgrounds, which suggest the value of improvement in access and training of culturally safe practices and tailoring the delivery of care to meet their needs.

The limitations of our study include the heterogeneity of our sample, which included different dementia subtypes, including those with unspecified dementia. However, this is an inherent limitation of conducting research in this context due to the lack of formal diagnosis of dementia process in Australian RAC,^26^ a pattern that has also been observed in other countries.^27^ Care staff proxy rating of residents' neuropsychiatric symptoms and quality of life may also be considered a limitation. However, this method was intentionally chosen with consideration of the high prevalence of severe dementia in RAC settings, which could have compromised self‐reporting. Additionally, previous research indicates that there are discrepancies between caregiver and self‐rated measures for people with dementia.^28^ In our study, assessments were conducted with members of staff who knew each particular resident best in order to maximise the validity of the data.

The NPI scores among our residents were also relatively low (mean NPI‐Q total score = 10.7). However, this is consistent with other studies in the RAC settings,^29^ while studies involving community‐dwelling people with dementia have reported higher NPI scores.^6^ This difference may be partly explained by the properties of NPI as an assessment tool, which requires a proxy interview to complete. Professional care staff in RAC facilities and informal caregivers of community‐dwelling people with dementia may have different perceptions related to the severity of symptoms.^30^ Care staff may minimise the severity and distress caused by symptoms, accepting these as part of dementia and emphasising their professionalism in being able to care for residents with various neuropsychiatric symptoms.

## 5 CONCLUSIONS

We examined the baseline demographic and clinical characteristics of a prospective cohort of residents with dementia and depressive symptoms residing in private RAC facilities who were participants in MIDDEL, a multinational, cluster‐randomised controlled trial. Findings from our study provide a detailed characterisation of the care needs of residents with dementia. Such knowledge can increase understanding and awareness of the associations between dementia severity, depression, neuropsychiatric symptoms and quality of life, and can be used to inform care planning and to promote targeted, person‐centred interventions through further research and staff training. Future studies comparing the profile of residents with dementia in other RAC settings (i.e. public facilities), as well as international contexts, are warranted in order to determine the generalisability of our findings. This will have important implications for future planning of care models proactively targeting the social and emotional needs of people with dementia and will help to inform policy recommendations to optimise aged care services for dementia care, as recommended by the Australian Royal Commission into Aged Care.

## CONFLICTS OF INTEREST

No conflicts of interest declared.

## Data Availability

De‐identified datasets (participant codes and outcome scores) generated during and/or analysed during the MIDDEL trial will be stored in a publicly available repository (NSD ‐ Norwegian Centre for Research Data, https://www.nsd.no/en/).
